# Peripheral blood biomarkers in PD-1/PD-L1 immunotherapy: distinguishing predictive from prognostic biomarkers

**DOI:** 10.3389/fimmu.2026.1924506

**Published:** 2026-08-27

**Authors:** Xuejun Guo, Yanhui Wei, Zhaoxu Miao, Shuhan Ma, Wenxue Ma

**Affiliations:** 1 Department of Hematology, Puyang Oilfield General Hospital Affiliated to Henan Medical University, Puyang, Henan, China; 2 Puyang Cell Therapy Engineering Technology Research Center, Puyang, Henan, China; 3 Department of Hematology, Zhongda Hospital, School of Medicine, Southeast University, Institute of Hematology Southeast University, Nanjing, China; 4 Sanford Stem Cell Institute, Department of Medicine, Moores Cancer Center, University of California, San Diego, San Diego, CA, United States

**Keywords:** cancer immunotherapy, immune monitoring, PD-1/PD-L1 blockade, peripheral blood biomarkers, predictive biomarkers, prognostic biomarkers

## Abstract

Immune checkpoint inhibitors targeting the programmed cell death protein 1 (PD-1) and programmed death ligand 1 (PD-L1) axis have transformed cancer therapy, yet reliable biomarkers for accurately predicting therapeutic benefit remain limited. Peripheral blood biomarkers have emerged as attractive candidates because of their minimally invasive accessibility, feasibility for serial monitoring, and potential to capture dynamic systemic immune responses during treatment. However, their clinical translation has been hindered by inconsistent findings, biological heterogeneity, and the frequent conflation of prognostic associations with true predictive value. Many commonly reported biomarkers, including circulating tumor DNA (ctDNA), blood-based tumor mutational burden (bTMB), inflammatory indices, and conventional serum markers, primarily reflect tumor burden, systemic inflammation, or host physiological status rather than genuine sensitivity to PD-1/PD-L1 blockade. In contrast, immune cell-derived biomarkers, particularly dynamic indicators of CD8^+^ T-cell activation and immune reinvigoration, offer stronger mechanistic relevance to checkpoint inhibitor responsiveness, although their predictive utility remains highly context dependent. In this review, we critically evaluate peripheral blood biomarkers using a mechanistically informed framework to distinguish predictive from prognostic biomarkers, integrating evidence from tumor-derived biomarkers, immune cell phenotyping, soluble inflammatory mediators, emerging immune repertoire and extracellular vesicle (EV) biomarkers, and composite longitudinal models. We further discuss translational barriers, clinical implementation challenges, and future priorities for biomarker development, aiming to provide a more rigorous framework for biomarker-guided precision immunotherapy.

## Highlights

- Prognostic biomarkers describe outcomes, whereas predictive biomarkers identify treatment-specific benefit
- Baseline tumor-burden and inflammatory markers are primarily prognostic and should not independently guide treatment selection
- Early ctDNA and immune-cell changes are promising pharmacodynamic tools for monitoring PD-1/PD-L1 blockade
- Composite models should integrate complementary, biologically relevant, and nonredundant tumor, immune, and host signals
- Clinical adoption requires standardized assays, independent validation, and evidence that biomarker-guided decisions improve patient outcomes

## 1 Introduction

Immune checkpoint blockade targeting the programmed cell death protein 1 (PD-1) and programmed death ligand 1 (PD-L1) axis has transformed the therapeutic landscape across multiple solid and hematologic malignancies by enabling durable antitumor immune responses in a subset of patients (1, 2). However, despite these advances, the majority of patients either fail to respond or eventually develop acquired resistance, highlighting the urgent need for reliable biomarkers to improve patient selection, guide treatment monitoring, and inform adaptive therapeutic strategies (3, 4). Currently approved biomarkers, including tumor PD-L1 expression, tumor mutational burden (TMB), and microsatellite instability (MSI), are predominantly tissue based and provide only imperfect predictive accuracy (5, 6). Their clinical utility is further constrained by intratumoral heterogeneity, temporal evolution during therapy, sampling bias, and the practical limitations of repeated tissue acquisition (7–10).

Against this backdrop, peripheral blood biomarkers have emerged as attractive alternatives because of their minimally invasive accessibility, feasibility for serial assessment, and potential to capture dynamic systemic immune and tumor-related changes during treatment (11, 12). Over the past decade, a rapidly expanding body of literature has explored diverse blood-based biomarkers including ctDNA, circulating tumor cells (CTCs), immune cell subsets, inflammatory indices, soluble cytokines, and other circulating molecular signatures as potential tools for predicting response to PD-1/PD-L1 blockade (13–15). Despite this enthusiasm, clinical interpretation remains inconsistent, and translational progress has been limited (16, 17).

A major conceptual challenge lies in the frequent conflation of prognostic and predictive biomarker functions (18, 19). Many peripheral blood biomarkers correlate with survival outcomes, tumor burden, or host physiological status regardless of treatment modality, yet are often interpreted as predictors of immunotherapy responsiveness (12, 20). This distinction is critical rather than semantic: prognostic biomarkers reflect the natural course or aggressiveness of disease, whereas predictive biomarkers indicate the likelihood of benefit from a specific therapeutic intervention (21, 22). Failure to clearly separate these roles has contributed to contradictory findings, inconsistent threshold definitions, and limited clinical adoption (23, 24).

Although several reviews have cataloged peripheral blood biomarkers associated with PD-1/PD-L1 inhibitor outcomes, fewer have critically examined the biological basis underlying these associations or addressed why many apparently promising biomarkers fail to translate into clinically actionable decision-making tools (12, 25). In particular, systemic blood-derived signals often reflect overlapping processes including tumor burden, systemic inflammation, immune competence, and treatment-induced perturbations making interpretation inherently complex.

In this review, we adopt a mechanistically informed and translational perspective to distinguish true predictive signals from prognostic noise among peripheral blood biomarkers in PD-1/PD-L1 immunotherapy (11, 26). Rather than providing a purely descriptive inventory, we evaluate biomarkers according to their biological origin, temporal dynamics, mechanistic relevance to immune checkpoint blockade, and potential clinical applicability (27). By doing so, we aim to clarify which circulating biomarkers may meaningfully support biomarker-guided precision immunotherapy, which primarily provide prognostic context, and how emerging composite and longitudinal strategies may help overcome current limitations.

## 2 Conceptual framework for interpreting peripheral blood biomarkers

Peripheral blood biomarkers differ fundamentally from tissue-based biomarkers in both biological origin and interpretive scope (12). Whereas tumor tissue biomarkers primarily interrogate localized tumor-immune interactions within the tumor microenvironment (TME), circulating biomarkers reflect a broader systemic landscape shaped by tumor burden, host immune competence, inflammatory tone, metabolic stress, and treatment-induced perturbations (28, 29). This complexity creates both opportunity and interpretive risk. A biologically grounded conceptual framework is therefore essential to distinguish clinically meaningful predictive signals from nonspecific prognostic associations. In this section, we outline three core principles that inform rational interpretation of peripheral blood biomarkers in PD-1/PD-L1 immunotherapy: the distinction between predictive and prognostic biomarker functions, the biological dimensions represented by circulating biomarkers, and the importance of temporal context in static versus dynamic biomarker assessment.

### 2.1 Predictive vs prognostic biomarkers: why the distinction matters

A fundamental challenge in the interpretation of peripheral blood biomarkers in PD-1/PD-L1 immunotherapy is the frequent failure to distinguish prognostic associations from true predictive utility (30, 31). Although these terms are often used interchangeably in the biomarker literature, they represent fundamentally different clinical concepts with distinct implications for therapeutic decision-making (32). Prognostic biomarkers provide information about the natural course, aggressiveness, or expected outcome of disease regardless of treatment, whereas predictive biomarkers identify the likelihood of benefit from a specific therapeutic intervention (22, 33). This distinction is particularly critical in immuno-oncology, where systemic biomarkers are often influenced by multiple overlapping biological processes unrelated to checkpoint inhibitor sensitivity (33, 34).

Many commonly reported peripheral blood biomarkers including inflammatory indices, serum tumor markers, lactate dehydrogenase (LDH), and even baseline ctDNA levels consistently correlate with overall survival (OS) or disease progression (20, 35, 36). However, such associations do not necessarily indicate predictive relevance for PD-1/PD-L1 blockade, as similar patterns are frequently observed across chemotherapy, targeted therapy, and supportive care settings (37–39). In these contexts, elevated biomarker levels may simply reflect advanced disease burden, compromised host physiological reserve, or chronic systemic inflammation rather than treatment-specific resistance. Misclassification of prognostic biomarkers as predictive tools risks inappropriate patient stratification and may partly explain the disappointing translational performance of many proposed biomarkers (40, 41).

The challenge is further amplified by the biological complexity of systemic circulation. Unlike tumor tissue biomarkers, which directly interrogate local tumor-immune interactions, peripheral blood biomarkers capture a composite systemic snapshot integrating tumor shedding, immune activation, inflammatory tone, metabolic stress, and treatment-induced perturbations (20, 42, 43). Consequently, the central question is not merely whether a biomarker correlates with clinical outcome, but whether the biological process it reflects is mechanistically linked to PD-1/PD-L1 therapeutic responsiveness (38, 44). Establishing this distinction provides the conceptual foundation for rational biomarker interpretation.

### 2.2 Biological dimensions of peripheral blood biomarkers

Peripheral blood biomarkers can be broadly interpreted across three interconnected biological dimensions: tumor burden and tumor-derived signals, immune competence and treatment-responsive immune activation, and systemic inflammation or immunosuppressive host states (45, 46). Recognizing these biological categories helps contextualize biomarker function and prevents overinterpretation of nonspecific associations.

The first dimension encompasses tumor-derived biomarkers, including ctDNA, bTMB, CTCs, and conventional serum tumor markers (14, 26, 47). These biomarkers primarily reflect disease burden, tumor kinetics, or molecular tumor characteristics (48, 49). Their clinical value is often strongest in prognostic stratification or treatment monitoring, particularly when evaluated longitudinally (50, 51). However, because tumor burden itself strongly influences survival outcomes independent of therapy type, baseline tumor-derived biomarkers may offer limited treatment-specific predictive precision (22, 52).

The second dimension includes immune cell-derived biomarkers, which are conceptually more relevant to PD-1/PD-L1 blockade because they interrogate the biological machinery directly targeted by immune checkpoint inhibition (2, 53). These include circulating CD8^+^ and CD4^+^ T-cell subsets, proliferative immune signatures, exhaustion phenotypes, immune repertoire dynamics, and other markers reflecting systemic immune readiness or reinvigoration (6, 54). Unlike tumor burden markers, these biomarkers may more directly capture whether immune checkpoint blockade can effectively restore antitumor immunity (53, 55). Nevertheless, interpretation remains highly dependent on timing, disease context, and treatment combinations (28, 56).

The third dimension comprises systemic inflammatory and immunosuppressive biomarkers, including neutrophil-to-lymphocyte ratio (NLR), C-reactive protein (CRP), cytokines such as IL-6 and IL-8, myeloid-derived suppressor cells (MDSCs), and metabolic stress indicators (57). These biomarkers often correlate strongly with poor outcomes, but their biological interpretation is more ambiguous (58, 59). Elevated inflammatory signals may indicate aggressive tumor biology, host frailty, chronic immunosuppression, or treatment-related immune perturbation rather than direct resistance to checkpoint blockade (60).

Importantly, these biological dimensions are not mutually exclusive. A single biomarker may simultaneously reflect multiple overlapping processes, reinforcing the need for mechanistic interpretation rather than simplistic classification. Accordingly, an integrative multi-biomarker or multi-omic approach that combines complementary tumor-derived, immune, and host-related signals may provide greater predictive resolution than evaluating each biomarker independently. However, such integration should be biologically guided and prioritize biomarkers that contribute nonredundant information. Indiscriminate aggregation of correlated features may amplify prognostic confounding, increase model complexity, and promote overfitting without meaningfully improving treatment-specific prediction.

### 2.3 Static versus dynamic biomarkers

Another critical conceptual distinction lies between static and dynamic biomarkers. A static biomarker is measured at a single prespecified time point most commonly before treatment-and interpreted without reference to its subsequent trajectory. In contrast, a dynamic biomarkers is defined by a within-patient change across at least two time points and may be characterized by its direction, magnitude, rate, or temporal pattern relative to treatment exposure (26, 61). Classification therefore depends on how the biomarker is measured and analyzed rather than on the analyte itself. For example, baseline ctDNA concentration is a static biomarker, whereas early ctDNA decline or clearance is dynamic. Similarly, a pretreatment extracellular vesicle (EV) or microRNA level is static, whereas an on-treatment change in EV cargo or circulating microRNA expression is dynamic.

Historically, biomarker development has focused heavily on pre-treatment measurements because of their appeal for patient selection (62). However, static baseline biomarkers often provide limited mechanistic resolution, as they are heavily influenced by disease burden, prior therapies, baseline inflammatory status, and host physiological variability (23).

In contrast, dynamic biomarkers may offer greater biological specificity because they capture treatment-induced changes in tumor behavior or immune activation (11, 61). For example, early ctDNA decline following PD-1/PD-L1 blockade may reflect therapeutic tumor control, whereas transient expansion of proliferating CD8^+^ T cells may indicate effective immune reinvigoration (4, 63). These dynamic responses are more directly linked to pharmacodynamic treatment effects than baseline static measurements. Circulating EVs and microRNAs may also function as dynamic biomarkers when serially assessed. Longitudinal changes in exosomal PD-L1, other immunoregulatory EV cargo, or circulating microRNA signatures may reflect evolving immune escape, tumor adaptation, or treatment-induced immune remodeling. However, their clinical evidence remains less mature than that supporting ctDNA kinetics because EV isolation methods, cargo normalization strategies, microRNA panels, analytical platforms, and sampling schedules remain poorly harmonized. Accordingly, EV- and microRNA-based dynamic biomarkers should currently be considered exploratory rather than clinically validated tools.

However, dynamic biomarkers also introduce practical and interpretive challenges (18). Optimal sampling windows remain incompletely standardized, biomarker kinetics may vary across tumor types and treatment regimens, and serial sampling increases logistical complexity (64, 65). Moreover, not all treatment-induced biomarker changes reflect beneficial antitumor immunity; some may instead signal systemic inflammation, immune-related toxicity, or transient immune perturbation (66, 67).

Combining baseline biomarkers with on-treatment changes is biologically attractive because the baseline component characterizes the pretreatment tumor–host state, whereas the dynamic component captures pharmacodynamic response. The DIREct-On model provides proof of concept by integrating pretreatment normalized bTMB and circulating CD8^+^ T-cell abundance with early ctDNA dynamics, achieving better discrimination of durable clinical benefit than any individual component (68). Nevertheless, combined models should not be presumed superior. Their incremental value must be demonstrated through prespecified comparisons of discrimination, calibration, and clinical net benefit in independent cohorts. Analyses must also address guarantee-time bias, because patients must remain alive and on treatment long enough to undergo the on-treatment assessment. Furthermore, although dynamic models may support early response monitoring or treatment adaptation, they cannot guide the initial treatment decision because the required measurements are obtained only after therapy has begun.

**Figure 1:**
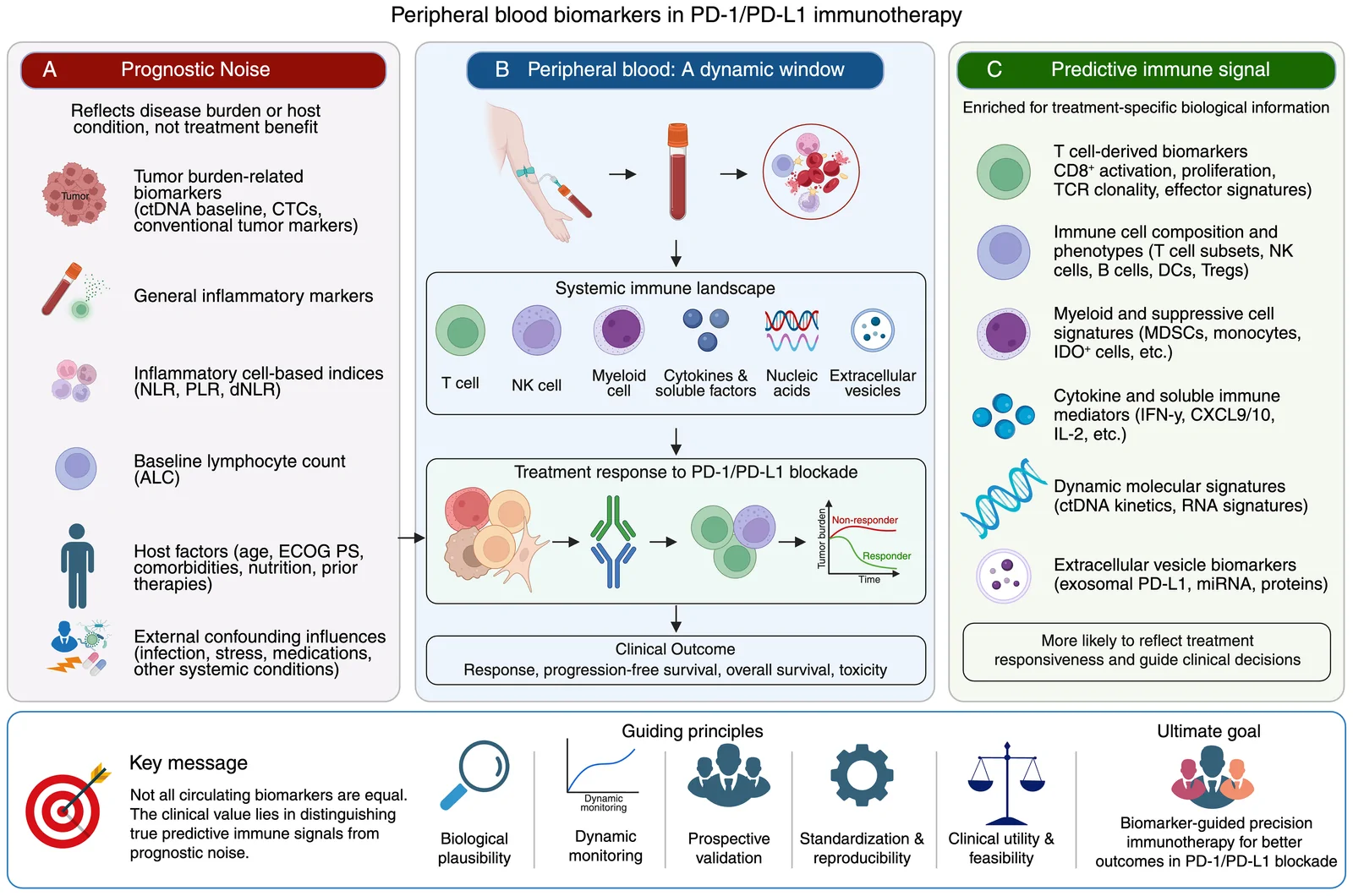
Conceptual framework for interpreting peripheral blood biomarkers in PD-1/PD-L1 immunotherapy. The three domains shown in the figure describe biomarker function and level of supporting evidence rather than mutually exclusive analyte classes. **(A)** Prognostic biomarkers provide information about clinical outcomes independent of a specific treatment and commonly reflect tumor burden, systemic inflammation, host physiological status, or external confounding factors. Examples include baseline ctDNA, conventional tumor markers, inflammatory indices, baseline lymphocyte counts, and host-related clinical factors. **(B)** Dynamic-window biomarkers are defined by serial within-patient measurements and capture treatment-associated changes in tumor-derived signals, immune cell populations, soluble mediators, nucleic acids, or extracellular vesicle (EV) cargo. These biomarkers primarily support pharmacodynamic monitoring and early response assessment but are not necessarily predictive of treatment-specific benefit. **(C)** Predictive biomarkers identify differential benefit from PD-1/PD-L1 blockade compared with an appropriate alternative treatment. Predictive status should ideally be supported by a prespecified treatment-by-biomarker interaction in a randomized study rather than by an outcome association within a single treatment cohort. Accordingly, the same analyte may occupy more than one domain depending on its biological context, timing, analytical approach, and supporting evidence. For example, baseline ctDNA is primarily prognostic, whereas early ctDNA clearance is a dynamic pharmacodynamic marker and becomes predictive only if it demonstrates differential treatment benefit. The lower panel summarizes the key requirements for clinical translation: biological plausibility, standardized longitudinal assessment, prospective validation, analytical reproducibility, and clinical utility.

Taken together, the distinction between static and dynamic biomarkers reinforces a broader principle: meaningful biomarker interpretation requires attention not only to biological origin, but also to temporal context. Integration of complementary baseline and longitudinal information may improve biomarker performance, provided that the added value is independently validated and aligned with a clearly defined clinical use. Given the biological complexity of circulating biomarkers, **Figure 1** presents the mechanistically informed framework used in this review to distinguish predictive immune signals from prognostic confounding influences in PD-1/PD-L1 immunotherapy.

## 3 Tumor-derived biomarkers: prognostic dominance with selective translational utility

Tumor-derived peripheral blood biomarkers, particularly those reflecting tumor burden, represent the most extensively studied category in immuno-oncology (69). Several tumor-associated peripheral blood biomarkers, including ctDNA, bTMB, CTCs, and conventional serum tumor markers, have been reported to correlate with survival and treatment outcomes (70, 71). However, their biological interpretation is often overstated. While these biomarkers robustly capture disease extent, tumor kinetics, and molecular characteristics, their ability to predict treatment-specific benefit from PD-1/PD-L1 blockade is fundamentally constrained (12, 16, 72). Understanding these limitations is essential for appropriately defining their clinical utility in biomarker-guided immunotherapy.

### 3.1 ctDNA: prognostic biomarker or early pharmacodynamic indicator?

Among tumor-derived blood biomarkers, ctDNA has emerged as the most clinically mature and widely investigated candidate in immuno-oncology (73, 74). Baseline ctDNA levels consistently correlate with tumor burden, metastatic dissemination, and adverse survival outcomes across multiple malignancies, making ctDNA a robust prognostic biomarker (75–77). However, elevated pre-treatment ctDNA does not necessarily indicate intrinsic resistance to PD-1/PD-L1 blockade, as similar prognostic associations are observed across chemotherapy, targeted therapy, and other systemic treatments (78, 79). In this setting, baseline ctDNA primarily answers the question of disease burden rather than immunotherapy sensitivity.

In contrast, longitudinal ctDNA kinetics provide a more biologically informative window into treatment response. Early reductions or clearance of ctDNA after initiation of PD-1/PD-L1 blockade have repeatedly been associated with improved progression-free and OS, suggesting utility as an early pharmacodynamic biomarker (80–82). Because these dynamic changes reflect treatment-induced tumor control rather than baseline disease characteristics, they may support adaptive clinical decision-making, particularly when radiographic interpretation is uncertain or delayed (83). Nevertheless, ctDNA dynamics remain fundamentally response-monitoring tools rather than pre-treatment predictive biomarkers, and their broader implementation remains constrained by assay variability, sensitivity limitations in low-shedding tumors, and lack of standardized timing frameworks (74, 84).

Prospective clinical evidence further supports the pharmacodynamic value of longitudinal ctDNA assessment. In the phase II INSPIRE study, which included patients with advanced solid tumors treated with pembrolizumab, a decrease in ctDNA by cycle 3 was associated with a higher objective response rate and longer progression-free and overall survival (85). These findings demonstrate that early ctDNA kinetics can provide clinically informative molecular-response assessment. However, because INSPIRE was a single-arm study without a non-immunotherapy comparator, it established ctDNA decline as an on-treatment pharmacodynamic and monitoring biomarker rather than a pretreatment predictor of pembrolizumab-specific benefit.

### 3.2 Blood-based tumor mutational burden: immunogenicity surrogate with biological limitations

Blood-based tumor mutational burden (bTMB) was developed as a minimally invasive alternative to tissue-derived TMB, based on the rationale that higher somatic mutational burden may increase neoantigen generation and thereby enhance susceptibility to immune checkpoint blockade (86, 87). Conceptually, this provides stronger predictive rationale than purely burden-related biomarkers. Indeed, selected studies have reported associations between elevated bTMB and improved outcomes with PD-1/PD-L1 inhibitors (88–90).

However, several biological and technical limitations complicate interpretation. First, accurate bTMB quantification depends heavily on ctDNA abundance, which itself is strongly influenced by tumor burden, metastatic distribution, and tumor shedding characteristics. This creates an inherent confounding relationship between immunogenicity assessment and disease burden (88, 89). Second, mutational quantity alone does not guarantee effective antitumor immunity. Successful response to checkpoint inhibition requires multiple complementary processes, including intact antigen presentation, functional interferon (IFN) signaling, T-cell priming, and effective immune infiltration (87, 91). Consequently, high bTMB may enrich for responsive populations at a cohort level while offering limited precision for individual treatment prediction (10, 92).

Additional translational barriers include assay heterogeneity, variable gene panel composition, inconsistent threshold definitions, and limited reproducibility across studies (89, 92). These limitations have prevented bTMB from achieving broad clinical adoption as a standalone biomarker. Thus, while bTMB retains conceptual relevance, its predictive value remains conditional rather than definitive (10, 90).

Landmark clinical trials illustrate both the initial promise and subsequent translational limitations of bTMB. Retrospective analyses of the randomized phase II POPLAR and phase III OAK trials identified a bTMB threshold of ≥16 mutations/Mb that enriched for progression-free survival benefit from atezolizumab versus docetaxel (93). However, subsequent prospective studies did not establish bTMB as a clinically reliable standalone selection biomarker. In the single-arm phase II B-F1RST trial, patients with bTMB ≥16 mutations/Mb showed numerical improvements in objective response rate and progression-free survival, but these associations did not reach the prespecified statistical significance thresholds (94). Similarly, the randomized phase III BFAST cohort C did not meet its primary progression-free survival endpoint for atezolizumab versus chemotherapy in patients with bTMB ≥16 mutations/Mb (95). In MYSTIC, exploratory analyses suggested improved overall survival with durvalumab plus tremelimumab in patients with bTMB ≥20 mutations/Mb; however, the overall trial did not meet its primary endpoints, and the exploratory bTMB threshold was not prospectively validated (96). Collectively, these studies demonstrate how retrospective cut-point selection, assay dependence, ctDNA-shedding bias, and insufficient treatment-by-biomarker interaction evidence can prevent a biologically plausible biomarker from achieving clinical utility.

### 3.3 CTCs: biological promise, translational inconsistency

CTCs offer another theoretically attractive blood-based biomarker because they provide direct access to viable tumor-derived material, potentially enabling both quantitative enumeration and phenotypic characterization. Elevated baseline CTC counts generally correlate with advanced disease burden, metastatic potential, and inferior survival outcomes across multiple cancer types (97–99). However, similar to ctDNA, these associations are predominantly prognostic rather than treatment specific (100, 101).

Efforts to enhance predictive relevance through molecular characterization of CTCs including PD-L1 expression profiling and genomic interrogation have yielded inconsistent results. Biological heterogeneity among CTC populations, limited assay standardization, low sensitivity in some tumor types, and technical variability in detection platforms have all hindered reproducibility (97, 102, 103). Moreover, PD-L1 expression on CTCs may not faithfully recapitulate intratumoral immune context or dynamic checkpoint biology (104, 105).

Although CTC-based analyses remain scientifically interesting and may contribute to future multimodal biomarker strategies, current evidence does not support their use as reliable standalone predictors of PD-1/PD-L1 therapeutic responsiveness.

### 3.4 Conventional serum tumor markers and LDH: accessible but biologically nonspecific

Conventional serum biomarkers including carcinoembryonic antigen (CEA), alpha-fetoprotein (AFP), cancer antigen family markers, and LDH remain among the most accessible circulating biomarkers in routine oncology practice. A growing body of evidence has linked elevated baseline levels to inferior outcomes following PD-1/PD-L1 blockade (20, 26). However, these biomarkers primarily reflect tumor burden, metabolic activity, tissue turnover, and systemic physiological stress rather than mechanisms directly related to immune checkpoint responsiveness (42).

LDH is particularly illustrative of this limitation. Elevated LDH frequently correlates with increased glycolytic tumor metabolism, hypoxia-associated adaptation, and inferior survival outcomes, yet similar prognostic associations have been reported across immunotherapy, chemotherapy, targeted therapy, and other treatment settings (106, 107). Likewise, conventional tumor markers may provide useful contextual information regarding disease kinetics but offer little mechanistic specificity for immunotherapy response prediction (102, 108).

Their principal clinical value therefore lies in baseline prognostic assessment and adjunctive disease monitoring rather than treatment selection. Reliance on such markers as predictive tools risks conflating poor prognosis with lack of immunotherapy benefit (12, 65).

Collectively, tumor-derived peripheral blood biomarkers provide clinically useful information regarding disease burden, molecular tumor characteristics, and treatment monitoring, but their capacity to predict treatment-specific benefit from PD-1/PD-L1 blockade remains limited (89). This distinction reinforces the broader conceptual framework outlined earlier: biomarkers that primarily reflect tumor burden generally provide prognostic context, whereas biomarkers derived from immune cell populations may offer greater predictive potential because they more directly capture the biological processes targeted by checkpoint inhibition.

## 4 Immune cell-derived biomarkers: potential predictive signals

In contrast to tumor-derived biomarkers, which predominantly reflect disease burden and tumor kinetics, immune cell-derived peripheral blood biomarkers offer closer mechanistic alignment with the biological processes directly targeted by PD-1/PD-L1 blockade (42, 56). Because immune checkpoint inhibitors function by restoring or amplifying antitumor immune responses, circulating immune cell states may provide more biologically meaningful insight into treatment responsiveness (109, 110). However, this promise is tempered by substantial complexity (56). Peripheral immune cell populations are highly dynamic, functionally heterogeneous, and shaped by tumor type, prior therapies, systemic inflammatory states, and treatment timing (111). Consequently, while immune cell-derived biomarkers may offer stronger predictive potential than tumor burden-related markers, their interpretation requires careful biological context (42).

### 4.1 Global immune indices (ALC, NLR, PLR)

Global immune indices derived from routine peripheral blood counts including absolute lymphocyte count (ALC), NLR, and platelet-to-lymphocyte ratio (PLR) are among the most frequently reported biomarkers in immunotherapy studies because of their simplicity, low cost, and widespread clinical availability. Across multiple tumor types, higher baseline lymphocyte counts and lower inflammatory ratios have consistently been associated with improved outcomes following PD-1/PD-L1 blockade (28, 112).

Despite this reproducibility, their predictive specificity remains limited. Similar associations have been observed in patients receiving chemotherapy, radiotherapy, targeted therapy, and even supportive care settings, indicating that these indices primarily reflect broader host physiological status rather than checkpoint-specific immune responsiveness (113, 114). Elevated NLR or PLR may indicate chronic systemic inflammation, immunosuppressive myeloid dominance, advanced disease burden, or reduced host immune reserve rather than intrinsic resistance to PD-1/PD-L1 blockade (113, 115, 116).

Interpretive challenges are further compounded by inconsistent cut-off definitions, inter-study heterogeneity, and limited biological granularity. Dynamic changes in these indices during therapy may provide additional prognostic information, but they remain insufficiently specific to function as standalone predictive biomarkers (117). Their greatest clinical value therefore lies in baseline contextual risk assessment rather than treatment selection.

### 4.2 CD8^+^ T-cell activation, proliferation, and exhaustion

Among circulating immune biomarkers, CD8^+^ T-cell populations offer perhaps the strongest mechanistic rationale for predictive relevance because they represent the principal effector cells reinvigorated by PD-1/PD-L1 blockade (28, 56). Unlike global inflammatory indices, functional states within CD8^+^ T-cell populations may more directly reflect immune readiness, treatment responsiveness, and adaptive resistance.

Several studies have demonstrated that early expansion of proliferating CD8^+^ T cells particularly Ki-67^+^ PD-1^+^ subsets following treatment initiation correlates with favorable responses to checkpoint inhibition (28, 56, 118). These dynamic changes likely reflect pharmacodynamic immune reinvigoration rather than baseline prognostic disease status, making them biologically more compelling than static pre-treatment biomarkers (119, 120). Similarly, shifts in effector memory differentiation or activation-associated phenotypes may provide insight into evolving antitumor immune engagement.

However, not all CD8^+^ T-cell signatures predict benefit. Terminal exhaustion, characterized by sustained inhibitory receptor expression, impaired proliferative capacity, metabolic dysfunction, and reduced effector competence, has been associated with therapeutic resistance (28, 121, 122). Yet even this interpretation is nuanced. Expression of inhibitory markers such as PD-1 alone does not necessarily indicate irreversible dysfunction, as activated tumor-reactive T cells may transiently express similar phenotypes (123, 124).

Thus, predictive interpretation depends less on simple enumeration and more on functional immune state, temporal dynamics, and phenotypic resolution. While CD8^+^ T-cell biomarkers remain among the most promising circulating predictive candidates, standardization and prospective validation remain essential.

### 4.3 CD4^+^ T cell subsets and regulatory T cells (Tregs)

CD4^+^ T-cell populations including helper T-cell subsets and Tregs play critical roles in shaping antitumor immunity and modulating checkpoint inhibitor responsiveness (117, 125). However, their interpretation as peripheral biomarkers is considerably more complex than that of CD8^+^ T cells because of substantial functional heterogeneity.

Activated helper CD4^+^ T cells may support durable antitumor immunity through cytokine production, dendritic cell licensing, and maintenance of cytotoxic T-cell responses (126). Conversely, Tregs suppress immune activation and may contribute to therapeutic resistance through immunosuppressive signaling and maintenance of peripheral tolerance. Accordingly, several studies have associated elevated circulating Treg frequencies with inferior outcomes (47, 127).

However, these relationships are not universally consistent. Treg expansion may also occur as a compensatory response to effective immune activation rather than primary resistance (128, 129). Similarly, peripheral abundance of helper T-cell populations does not necessarily mirror intratumoral functional activity. Phenotypic overlap between activated and suppressive subsets further complicates interpretation, particularly when relying on limited surface marker definitions (118).

As a result, simple quantification of CD4^+^ subsets provides limited predictive precision. Greater mechanistic resolution including activation state, functional phenotype, and longitudinal assessment will likely be necessary before these biomarkers can be reliably integrated into clinical decision-making (130).

### 4.4 MDSCs and monocyte signatures

Beyond lymphocyte populations, circulating myeloid compartments have emerged as important contributors to immunotherapy response and resistance. MDSCs, suppressive monocyte subsets, and dysfunctional antigen-presenting populations may profoundly shape systemic immune competence and influence responsiveness to PD-1/PD-L1 blockade (131, 132).

Elevated circulating MDSC levels have frequently been associated with poor outcomes, consistent with their capacity to suppress T-cell activation, impair antigen presentation, promote tumor-associated inflammation, and sustain immunosuppressive microenvironments (133, 134). Similarly, monocyte phenotypes characterized by reduced antigen-presenting capacity or immunoregulatory transcriptional programs may indicate ineffective systemic immune priming.

These biomarkers are conceptually attractive because they reflect biologically relevant mechanisms of immune suppression rather than merely generalized host inflammation. However, translational barriers remain substantial. MDSC definitions vary considerably across studies, phenotypic classification lacks standardization, and functional assays are often not readily a to routine clinical workflows. Monocyte transcriptional or phenotypic signatures similarly remain exploratory (133, 135).

Collectively, immune cell-derived peripheral blood biomarkers offer greater mechanistic relevance to PD-1/PD-L1 therapeutic responsiveness than tumor-derived or purely inflammatory biomarkers (42, 118). As understanding of systemic myeloid immunobiology continues to advance, biomarkers derived from suppressive myeloid populations may become increasingly important components of integrated predictive models (136). However, the predictive potential of immune cell-derived biomarkers depends heavily on biological resolution, functional interpretation, and temporal context (137). Static immune cell counts alone are rarely sufficient; rather, meaningful predictive insight increasingly emerges from dynamic immune state assessment (138). This complexity becomes even more evident when considering soluble inflammatory mediators, which often blur the boundary between immune activation, systemic stress, and treatment-related toxicity (42).

Given the heterogeneity of circulating biomarkers and their differing biological implications, **Table 1** provides a comparative framework distinguishing biomarker with primarily prognostic utility from those with greater predictive potential in PD-1/PD-L1 immunotherapy.

**Table 1:** Established peripheral blood biomarkers in PD-1/PD-L1 immunotherapy: distinguishing predictive potential from prognostic association.

Biomarker	Biological rationale	Primary clinical implication	Predictive relevance	Key limitations	References
Circulating tumor DNA (ctDNA)	Reflects tumor burden, molecular tumor shedding, treatment-induced tumor clearance	Prognostic assessment; early treatment monitoring	Moderate (dynamic); low (baseline)	Baseline values largely reflect disease burden; assay variability; limited sensitivity in low-shedding tumors	(80, 139)
Blood-based tumor mutational burden (bTMB)	Surrogate for neoantigen load and tumor immunogenicity	Exploratory patient enrichment	Moderate but context dependent	Strong dependence on ctDNA abundance; assay heterogeneity; inconsistent thresholds	(86, 140)
Circulating tumor cells (CTCs)	Reflect tumor dissemination and viable tumor biology	Prognostic stratification; exploratory molecular characterization	Low	Low sensitivity; biological heterogeneity; limited reproducibility	(141, 142)
Conventional serum tumor markers (CEA, AFP, CA family)	Reflect tumor burden, tumor turnover, disease kinetics	Disease monitoring; prognostic context	Low	Limited treatment specificity; tumor-type dependency	(12, 42)
Lactate dehydrogenase (LDH)	Reflects tumor metabolism, hypoxia, cellular turnover	Prognostic risk stratification	Low	Nonspecific; influenced by multiple non-tumor factors	(113, 143)
Absolute lymphocyte count (ALC)	Surrogate of systemic immune competence	Baseline host immune context	Low	Limited biological specificity; influenced by prior therapies and comorbidities	(42, 118)
Neutrophil-to-lymphocyte ratio (NLR)	Reflects inflammatory tone and immune suppression	Prognostic contextual biomarker	Low	Variable cutoffs; nonspecific systemic inflammatory signal	(114, 144)
Platelet-to-lymphocyte ratio (PLR)	Reflects inflammatory and thrombo-inflammatory host state	Prognostic contextual biomarker	Low	Limited specificity; inconsistent validation	(115, 144)
CD8^+^ T-cell activation/proliferation signatures	Directly reflects immune reinvigoration under PD-1 blockade	Early pharmacodynamic monitoring; predictive exploration	High (dynamic)	Timing sensitivity; phenotypic heterogeneity; assay standardization challenges	(28, 118)
CD4^+^ helper/regulatory T-cell subsets	Reflect immune support versus immunosuppression	Exploratory immune monitoring	Moderate but inconsistent	Functional heterogeneity; poor marker standardization	(118, 145)
MDSCs/suppressive monocyte signatures	Reflect systemic immune suppression and impaired antigen presentation	Investigational resistance biomarkers	Moderate (emerging)	Variable phenotypic definitions; functional assay complexity	(133, 134)
IL-6/IL-8/inflammatory cytokines	Reflect inflammatory immune dysregulation	Risk stratification; mechanistic context	Low to moderate	Poor specificity; overlap with toxicity, infection, systemic stress	(42, 146)
CRP/ferritin/acute-phase reactants	Reflect systemic inflammatory activation	Prognostic context	Low	Highly nonspecific	(114, 147)
TCR repertoire dynamics	Reflect adaptive antitumor immune activation	Emerging predictive biomarker	High potential	Bioinformatic complexity; incomplete validation	(148, 149)

## 5 Soluble inflammatory and immune mediators: informative but nonspecific

Soluble mediators measurable in peripheral blood, including cytokines, chemokines, acute-phase reactants, and metabolic biomarkers, have long been investigated as readily accessible indicators of systemic immune and inflammatory states in patients with cancer (12, 150). In the context of PD-1/PD-L1 immunotherapy, these circulating factors are particularly appealing because they may reflect dynamic immune activation, inflammatory responses, or treatment-induced perturbations without requiring complex cellular profiling (105, 118). However, their biological interpretation is inherently challenging. Unlike immune cell-derived biomarkers that may directly interrogate functional immune states, soluble mediators often reflect overlapping processes including tumor burden, chronic inflammation, metabolic stress, infection, immune-related toxicity, and host physiological status (114, 118). As a result, many demonstrate prognostic value while offering limited treatment-specific predictive precision.

### 5.1 Pro-inflammatory cytokines: biomarkers of resistance or disease severity?

Among soluble immune mediators, cytokines such as interleukin-6 (IL-6), interleukin-8 (IL-8), tumor necrosis factor-alpha (TNF-α), and interferon-associated inflammatory signals have frequently been investigated as candidate biomarkers for checkpoint inhibitor response (151). Elevated baseline levels of IL-6 and IL-8 have repeatedly been associated with inferior response rates, shorter progression-free survival (PFS), and reduced OS across multiple tumor types (152, 153).

These observations are biologically plausible. IL-6 promotes chronic inflammatory signaling, immune dysregulation, myeloid expansion, and T-cell dysfunction, while IL-8 contributes to neutrophil recruitment, immunosuppressive myeloid activation, and tumor-promoting inflammatory remodeling (154). Such mechanisms provide conceptual rationale for linking elevated cytokine levels to immunotherapy resistance.

However, predictive interpretation remains complicated. Elevated cytokine concentrations frequently accompany advanced disease burden, cancer-associated cachexia, chronic systemic inflammation, infection, and other adverse host biological states unrelated to treatment responsiveness (155). Thus, whether these markers directly mediate checkpoint resistance or simply reflect aggressive systemic disease biology is often difficult to determine. Dynamic cytokine fluctuations during therapy may provide additional information, but interpretation remains similarly ambiguous, as transient inflammatory elevations may signal either productive immune activation or emerging toxicity (156).

Accordingly, pro-inflammatory cytokines should be interpreted as biologically informative but context-dependent biomarkers rather than definitive standalone predictors (12, 42).

### 5.2 Acute-phase reactants and metabolic stress biomarkers

Acute-phase reactants and metabolic biomarkers including CRP, LDH, ferritin, and related inflammatory markers are particularly attractive because of their widespread clinical availability, low cost, and ease of implementation (157). Elevated baseline CRP and LDH levels have consistently been associated with inferior outcomes in patients receiving PD-1/PD-L1 inhibitors (158).

Nevertheless, these biomarkers primarily reflect systemic inflammatory burden, tumor metabolism, tissue turnover, hypoxia, or generalized physiological stress rather than mechanisms specifically linked to immune checkpoint responsiveness (159). CRP serves as a downstream marker of inflammatory cytokine signaling, whereas LDH reflects aggressive tumor metabolism and cellular turnover (160). Similar prognostic associations are frequently observed across non-immunotherapy treatment settings, reinforcing their limited predictive specificity.

These markers may therefore contribute meaningfully to baseline risk stratification or contextual clinical interpretation but should not be viewed as treatment-selection biomarkers in isolation. Their greatest utility may emerge when integrated with more mechanistically specific immune or tumor-derived biomarkers (42, 118).

### 5.3 Distinguishing therapeutic immune activation from immune-related toxicity

A particularly challenging aspect of soluble biomarker interpretation is the overlap between biomarkers associated with therapeutic immune activation and those associated with immune-related adverse events (irAEs) (161, 162). Because checkpoint blockade functions by enhancing immune activation, transient elevations in inflammatory mediators may accompany effective antitumor responses (163). However, similar biomarker changes may also precede pathological immune toxicity without durable clinical benefit.

This interpretive overlap limits the specificity of soluble biomarkers. Elevated cytokines or acute-phase markers may reflect beneficial immune reinvigoration, excessive inflammatory toxicity, uncontrolled tumor progression, or combinations of these processes (164). Without integration with cellular immune biomarkers, tumor-derived signals, or temporal clinical context, isolated soluble marker measurements provide limited mechanistic clarity (69).

This distinction is particularly relevant in clinical translation, where misinterpretation could lead to inappropriate escalation, discontinuation, or premature therapeutic modification.

Collectively, soluble inflammatory and immune mediators provide valuable insight into systemic biological context, but their interpretive limitations constrain their utility as standalone predictive biomarkers for PD-1/PD-L1 blockade (165). Their greatest value may lie in contextual risk assessment, mechanistic hypothesis generation, or incorporation into multidimensional biomarker models rather than independent clinical decision-making tools (166). These limitations underscore the need to explore emerging biomarker platforms capable of capturing more specific immune and tumor biology with greater mechanistic resolution.

## 6 Emerging peripheral blood biomarkers and next-generation technologies

The limitations of conventional peripheral blood biomarkers have driven increasing interest in next-generation biomarker platforms capable of providing deeper mechanistic resolution and greater predictive specificity (11, 22). Emerging approaches increasingly move beyond simple enumeration of circulating tumor burden or broad inflammatory indices toward multidimensional characterization of systemic immune states, tumor-immune interactions, and treatment-induced biological adaptation (167). These technologies including immune repertoire profiling, EV analysis, circulating transcriptomic signatures, and high-dimensional single-cell immune phenotyping offer the potential to capture biological processes more directly relevant to checkpoint inhibitor responsiveness (168). However, despite substantial promise, most remain investigational and face important translational barriers related to standardization, interpretability, cost, and clinical implementation (169).

### 6.1 T-cell receptor repertoire biomarkers

TCR repertoire profiling has emerged as a biologically compelling strategy for assessing systemic antitumor immune readiness and treatment responsiveness (170). Because effective PD-1/PD-L1 blockade depends on reactivation of tumor-reactive T-cell populations, metrics such as TCR clonality, diversity, clonal expansion, and treatment-induced repertoire remodeling may provide more direct mechanistic insight than conventional bulk immune cell counts (28).

Several studies have suggested that expansion of specific peripheral T-cell clones following checkpoint inhibition may correlate with therapeutic response, reflecting successful immune reinvigoration and antigen-driven activation (171). Conversely, highly restricted repertoires or failure to demonstrate adaptive clonal remodeling may indicate limited immune responsiveness. Dynamic TCR monitoring may therefore offer greater predictive relevance than static baseline immune phenotyping (149, 172).

However, interpretation remains complex. Expanded peripheral clones are not necessarily tumor reactive, repertoire diversity metrics may vary depending on analytical methodology, and relationships between peripheral and intratumoral T-cell dynamics remain incompletely defined (173). Technical variability, bioinformatic complexity, and lack of standardized analytical frameworks currently limit broader clinical applicability. Nevertheless, immune repertoire profiling remains one of the most mechanistically attractive emerging biomarker strategies in immuno-oncology (174).

### 6.2 Extracellular vesicle and exosomal biomarkers

Extracellular vesicles (EVs), including exosomes, have gained increasing attention as minimally invasive biomarker platforms because they carry molecular cargo reflective of both tumor and immune-cell states (175). These vesicles contain proteins, nucleic acids, lipids, and immunoregulatory molecules capable of influencing systemic immune function and tumor-immune communication (176).

Particularly relevant to PD-1/PD-L1 immunotherapy, exosomal PD-L1 has been proposed as a candidate biomarker of immune evasion and treatment resistance (177). Elevated circulating exosomal PD-L1 levels have been associated in some studies with poor therapeutic response, potentially reflecting active systemic immunosuppressive signaling (177, 178). Similarly, EV-associated microRNAs, messenger RNAs, and immune regulatory proteins may provide insight into tumor adaptation, immune escape, and treatment-induced biological remodeling (179).

Despite these intriguing findings, translational challenges remain substantial. EV isolation methodologies vary considerably, molecular cargo characterization lacks standardization, and biological interpretation is complicated by mixed cellular origins (180). Moreover, reproducibility across studies remains limited. At present, EV biomarkers are best viewed as promising exploratory tools rather than clinically actionable predictive biomarkers (176, 181).

### 6.3 Circulating microbial DNA and microbiome-derived metabolites

The gut microbiome may influence systemic responsiveness to PD-1/PD-L1 blockade through microbial products that enter the circulation and modulate antigen-presenting cells, T-cell metabolism, and inflammatory signaling (182, 183). Candidate blood-accessible biomarkers include microbial cell-free DNA and microbiome-derived metabolites such as short-chain fatty acids (SCFAs) and inosine (182, 183). In patients with solid tumors treated with nivolumab or pembrolizumab, higher concentrations of fecal and plasma SCFAs were associated with longer progression-free survival, supporting a potential relationship between microbial metabolism and checkpoint inhibitor efficacy (182). Preclinical studies have further demonstrated that microbiome-derived inosine can enhance the response to checkpoint blockade by promoting antitumor T-cell activity under appropriate immunological conditions (184).

Proof-of-principle clinical studies in anti-PD-1-refractory melanoma have also shown that fecal microbiota transplantation followed by anti-PD-1 reinduction can modify the gut microbiome and restore clinical responses in a subset of patients (185, 186). However, these findings do not establish circulating microbial DNA or metabolites as validated predictive biomarkers. Microbial cell-free DNA analysis is particularly susceptible to low-biomass contamination and batch effects (187), while circulating metabolite concentrations may be influenced by diet, antibiotic exposure, geography, comorbidities, and host metabolism (188, 189). These biomarkers should therefore remain exploratory until standardized preanalytical procedures, contamination controls, reproducible thresholds, longitudinal sampling frameworks, and prospective treatment-interaction testing are established.

### 6.4 Circulating transcriptomic and gene expression signatures

Peripheral blood transcriptomic profiling offers another emerging avenue for biomarker discovery by capturing global immune activation states, inflammatory programs, IFN signaling, and myeloid or lymphoid immune dynamics (190). Unlike single-marker approaches, transcriptomic signatures may better reflect coordinated biological programs relevant to checkpoint inhibitor responsiveness (191).

Several studies have explored peripheral blood RNA signatures associated with favorable or unfavorable immunotherapy outcomes, including IFN response programs, T-cell activation signatures, inflammatory gene modules, and myeloid suppression-related expression patterns (164, 192). These approaches may offer greater biological depth than routine immunophenotyping while remaining compatible with minimally invasive serial monitoring.

However, transcriptomic biomarkers also present interpretive challenges. Gene expression signatures may reflect mixed cell populations, inflammatory noise, prior treatment effects, or systemic comorbid biological processes (193, 194). Signature reproducibility across disease contexts remains variable, and analytical complexity may limit routine clinical implementation (195). Nevertheless, circulating transcriptomic profiling represents an important bridge between conventional biomarker assessment and systems-level immune monitoring (196).

### 6.5 High-dimensional single-cell immune profiling

Advances in high-dimensional immune profiling technologies including spectral flow cytometry, mass cytometry (CyTOF), and single-cell RNA sequencing (scRNA-seq) have transformed understanding of systemic immune heterogeneity in cancer immunotherapy (197, 198). These approaches allow detailed characterization of immune cell phenotypes, functional states, differentiation trajectories, exhaustion programs, and treatment-induced immune remodeling at unprecedented resolution (199).

In the context of PD-1/PD-L1 blockade, single-cell approaches have identified circulating immune signatures associated with response, resistance, and toxicity, including proliferative CD8^+^ T-cell subsets, dysfunctional exhaustion programs, suppressive myeloid states, and dynamic shifts in immune trajectories during therapy (110). Compared with conventional bulk biomarkers, these technologies offer substantially greater mechanistic specificity and may help distinguish biologically meaningful predictive signals from nonspecific systemic noise (200).

Yet significant translational barriers remain. High cost, technical complexity, specialized bioinformatics requirements, inter-platform variability, and challenges in clinical standardization currently restrict widespread implementation (201). Furthermore, not all highly granular biological signatures will ultimately prove clinically actionable. Nevertheless, these technologies are likely to play a central role in future biomarker discovery and mechanistic validation (202).

Collectively, next-generation peripheral blood biomarker platforms offer substantially greater mechanistic depth than conventional circulating biomarkers and may help overcome some of the interpretive limitations that have hindered clinical translation (11). However, increased biological resolution does not automatically confer clinical utility. The ultimate challenge lies in integrating mechanistically informative biomarkers into practical predictive frameworks that remain reproducible, interpretable, and operationally feasible (18). This challenge has driven growing interest in composite and longitudinal biomarker models that combine complementary biological dimensions rather than relying on single-marker prediction (203).

To summarize the emerging biomarker platforms that may overcome the limitations of conventional circulating biomarkers, **Table 2** highlights next-generation technologies, their biological rationale, potential translational applications, and major implementation barriers.

**Table 2:** Emerging peripheral blood biomarker platforms and next-generation technologies for PD-1/PD-L1 immunotherapy.

Emerging biomarker platform	Biological focus	Potential application	Key barriers	References
TCR repertoire profiling	Adaptive immune diversity and clonal expansion	Response prediction; immune monitoring	Bioinformatic complexity; limited standardization	(149, 204)
Extracellular vesicle/exosomal biomarkers	Tumor immune communication; immune evasion	Early resistance detection; dynamic monitoring	Isolation variability; mixed cellular origins	(205, 206)
Circulating transcriptomic signatures	Immune activation and inflammatory programs	Immune stratification; treatment monitoring	Signature heterogeneity; reproducibility concerns	(150, 207)
Peripheral blood scRNA-seq	Single-cell immune state mapping	Biomarker discovery; resistance profiling	High cost; technical complexity	(208, 209)
Mass cytometry (CyTOF)	High-dimensional immune phenotyping	Mechanistic immune monitoring	Specialized infrastructure; limited scalability	(210, 211)
Spectral flow cytometry	Translational immunophenotyping	Prospective immune monitoring	Panel harmonization challenges	(12, 118)
Multi-omic integration	Combined tumor-immune biological profiling	Composite biomarker modeling	Integration complexity; validation burden	(11, 211)
Machine learning (ML) frameworks	Multidimensional predictive modeling	Personalized prediction support	Overfitting; limited interpretability	(11, 212)
Circulating microbial DNA and microbiome-derived metabolites	Systemic microbial–immune modulation	Exploratory response stratification and treatment monitoring	Low-biomass contamination; dietary, antibiotic, geographic, and metabolic confounding	(182, 187)

**Figure 2:**
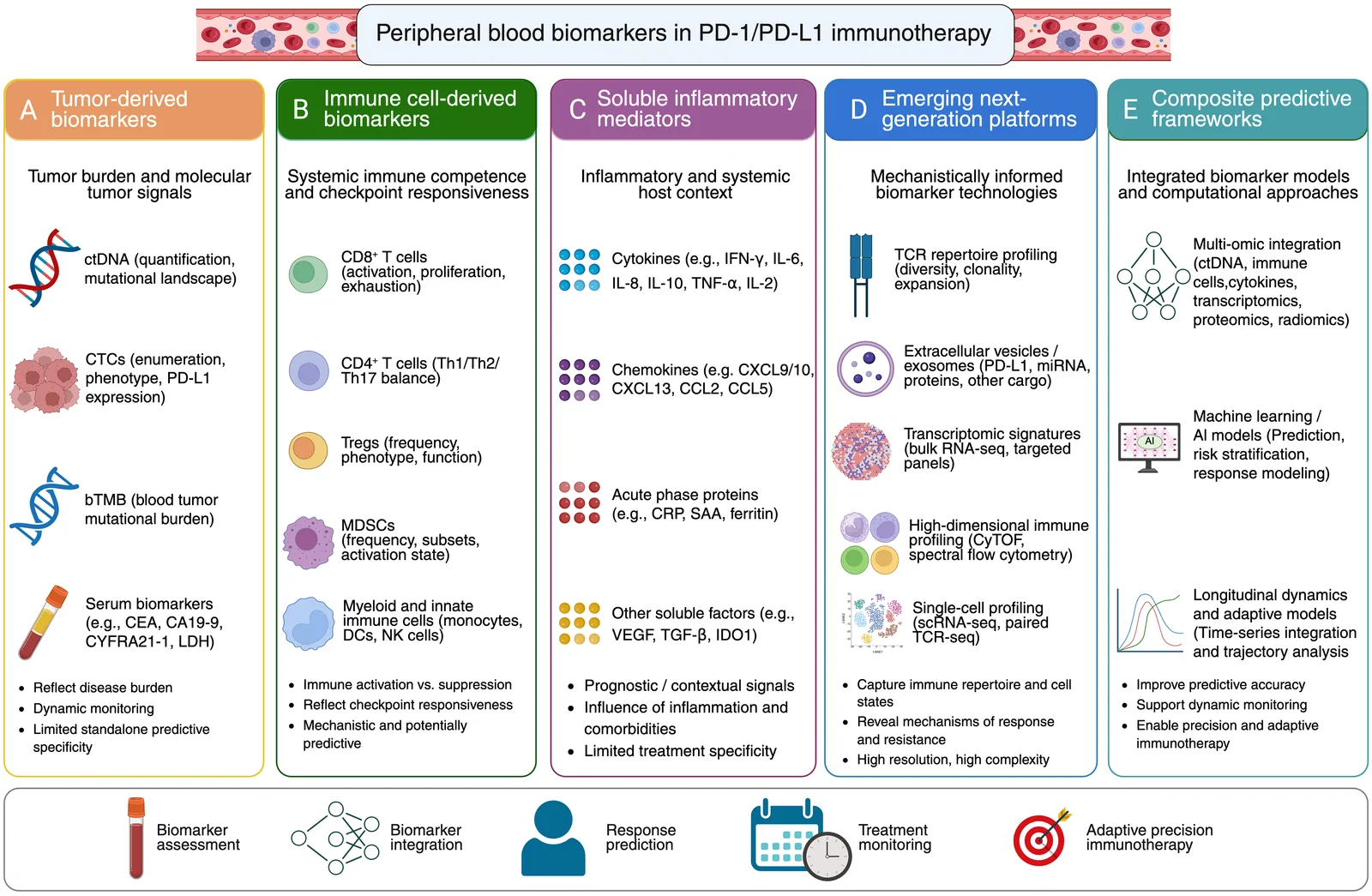
Classification of peripheral blood biomarker platforms in PD-1/PD-L1 immunotherapy. Peripheral blood biomarkers used to evaluate response to PD-1/PD-L1 blockade encompass multiple complementary biological dimensions. **(A)** Tumor-derived biomarkers, including ctDNA, CTCs, blood tumor mutational burden (bTMB), and serum biomarkers, primarily reflect tumor burden, molecular tumor characteristics, and disease kinetics. **(B)** Immune cell-derived biomarkers provide insight into systemic immune competence and checkpoint responsiveness through the assessment of effector, regulatory, and suppressive immune cell populations. **(C)** Soluble inflammatory mediators, including cytokines, chemokines, acute-phase proteins, and other circulating immunoregulatory factors, capture the broader inflammatory and host physiological context that influences treatment outcomes. **(D)** Emerging next-generation biomarker platforms, such as TCR repertoire profiling, EV analysis, transcriptomic profiling, and high-dimensional immune phenotyping, offer enhanced biological resolution and mechanistic insight into response and resistance. **(E)** Composite predictive frameworks integrate multidimensional biomarker data through multi-omic approaches, ML algorithms, and longitudinal modeling to improve predictive performance and support adaptive, biomarker-guided precision immunotherapy. The lower panel illustrates the translational workflow from biomarker assessment and integration to response prediction, treatment monitoring, and adaptive precision immunotherapy.

To provide an integrated overview of the peripheral blood biomarker landscape, **Figure 2** summarizes the major biomarker categories and emerging platforms relevant to PD-1/PD-L1 immunotherapy.

## 7 Composite and longitudinal biomarker models

The limitations of individual peripheral blood biomarkers whether tumor-derived, immune cell-based, inflammatory, or emerging multi-omic platforms underscore a central reality in biomarker development for PD-1/PD-L1 immunotherapy: treatment responsiveness is governed by complex and dynamic biological interactions that cannot be adequately captured by single-marker approaches (11, 14). Tumor burden, immune competence, inflammatory tone, treatment-induced immune remodeling, and adaptive resistance mechanisms interact continuously throughout therapy (213). Consequently, growing attention has shifted toward composite and longitudinal biomarker frameworks that integrate complementary biological dimensions to improve predictive precision and clinical interpretability (150, 208).

### 7.1 Rationale for composite biomarker approaches

Composite biomarker strategies are based on the premise that clinically meaningful predictive signals emerge from integration rather than isolation. Individual biomarkers often capture only one biological dimension and are therefore vulnerable to misinterpretation (11). For example, ctDNA may provide insight into tumor burden or early treatment response but offers limited information regarding systemic immune readiness. Conversely, circulating CD8^+^ T-cell activation signatures may reflect immune reinvigoration yet fail to account for overwhelming disease burden, myeloid suppression, or systemic inflammatory constraints (214).

By integrating biomarkers that interrogate distinct, yet complementary biological processes such as tumor burden, immune activation, immune suppression, and systemic inflammatory context, composite models may better distinguish true immunotherapy responsiveness from nonspecific prognostic associations. However, not every biomarker included in a composite model provides independent information. Routine variables such as NLR, CRP, IL-6, albumin, LDH, and performance status may partially encode the same underlying processes, including systemic inflammation, tumor burden, cachexia, or reduced host physiological reserve (215). Combining highly correlated variables may therefore increase apparent model complexity without adding meaningful predictive information. Candidate biomarkers should be selected based on mechanistic relevance and evaluated for collinearity, redundancy, stability, and incremental predictive value (57).

A composite model should be considered genuinely improved only if it outperforms a parsimonious reference model and its individual components in independent validation cohorts (216). Evaluation should include discrimination, calibration, and decision-curve analysis to determine whether the model provides clinically meaningful net benefit across relevant decision thresholds (217). Improvements observed only in the development cohort may reflect overfitting rather than reproducible predictive value (218). Furthermore, when a composite model is proposed for treatment selection, randomized data should demonstrate a treatment-by-model interaction indicating differential benefit from PD-1/PD-L1 blockade compared with an appropriate alternative treatment. Without such evidence, the model may simply combine multiple prognostic factors rather than provide treatment-specific prediction (219). This multidimensional framework is conceptually aligned with the biological complexity of checkpoint inhibitor response and offers a more realistic path toward clinically actionable biomarker development (12, 150).

### 7.2 Longitudinal and dynamic biomarker frameworks

Temporal context represents a critical but often underappreciated dimension of biomarker interpretation. Static baseline biomarkers may provide useful prognostic context but frequently fail to capture the dynamic biological evolution induced by checkpoint blockade. In contrast, longitudinal biomarker monitoring enables assessment of treatment-induced changes in tumor control, immune reinvigoration, inflammatory adaptation, and emerging resistance (220).

Examples include early ctDNA clearance as an indicator of pharmacodynamic tumor response, transient expansion of proliferating CD8^+^ T-cell populations as evidence of immune activation and evolving inflammatory signatures that may signal resistance or toxicity (4, 84). Dynamic biomarker trajectories may therefore provide substantially greater biological specificity than isolated pre-treatment measurements (221).

However, implementation requires careful consideration of sampling timing, disease-specific kinetics, treatment regimen heterogeneity, and interpretation of transient fluctuations. Not all biomarker changes indicate durable therapeutic benefit; some may reflect temporary immune perturbation, inflammatory toxicity, or nonproductive immune activation (222). Nonetheless, longitudinal assessment represents one of the most promising strategies for improving biomarker relevance in immunotherapy (223).

### 7.3 Computational integration and ML-assisted prediction

The increasing complexity of multidimensional biomarker data has naturally driven interest in computational integration and ML-assisted predictive modeling. These approaches offer the ability to identify nonlinear relationships, temporal interactions, and multidimensional biological patterns that may be difficult to resolve using conventional analytical methods (18, 224).

Potential applications include integrating ctDNA kinetics, immune cell phenotyping, transcriptomic signatures, inflammatory biomarkers, and clinical metadata into unified predictive models. Such approaches may improve individualized risk stratification, early response assessment, and adaptive therapeutic decision-making (202).

Two models provide notable examples of this approach. DIREct-On integrates pretreatment ctDNA-normalized bTMB, circulating CD8^+^ T-cell abundance, and early on-treatment ctDNA dynamics to identify patients with advanced NSCLC who are likely to achieve durable clinical benefit from immune checkpoint inhibition. The combined model demonstrated better performance than any individual component, supporting the value of integrating complementary baseline and dynamic biomarkers (68). More recently, SCORPIO integrated routine complete blood counts, metabolic laboratory measurements, and clinical variables from 9, 745 immune checkpoint inhibitor-treated patients across 21 cancer types. The model was evaluated in independent real-world cohorts and 10 global phase III clinical trials and demonstrated reproducible discrimination for clinical benefit and survival outcomes, outperforming PD-L1 expression and TMB in the analyzed datasets (225).

Nevertheless, these models remain investigational. DIREct-On requires broader prospective and multicenter validation, whereas SCORPIO may derive part of its performance from general prognostic variables rather than exclusively treatment-specific biology. Neither model has yet demonstrated in a prospective biomarker-guided impact trial that its use improves treatment selection or patient outcomes. Smaller single-center ML models based on serial blood counts, cytokines, or immune-cell subsets should likewise be considered hypothesis-generating until independently reproduced.

However, enthusiasm for computational prediction should be tempered by important limitations. Overfitting, limited external validation, insufficient biological interpretability, dataset heterogeneity, and poor reproducibility continue to constrain the clinical applicability of many proposed models (226, 227). Black-box models that achieve strong statistical performance without mechanistic transparency may be difficult to implement and validate in clinical practice. Accordingly, computational models should complement rather than replace biologically informed biomarker interpretation (228).

### 7.4 Translational barriers to implementation

Despite strong conceptual appeal, composite and longitudinal biomarker strategies face substantial translational barriers (12). Assay standardization remains inconsistent across ctDNA platforms, immune phenotyping methodologies, transcriptomic analyses, and emerging biomarker technologies (229). Optimal biomarker combinations remain undefined, and disease-specific biological variability further complicates generalizability (230).

Serial sampling requirements may increase logistical complexity, cost, and patient burden, while sophisticated analytical workflows may be difficult to implement outside specialized centers (231). Regulatory validation presents an additional challenge, particularly for composite models integrating multiple assays or adaptive computational frameworks (232).

Most importantly, many proposed models remain retrospective or exploratory. Prospective validation within well-designed clinical studies with predefined biomarker hypotheses, standardized sampling schedules, and clinically meaningful endpoints is essential before widespread adoption (233).

Collectively, composite and longitudinal biomarker models represent one of the most rational strategies for improving predictive precision in PD-1/PD-L1 immunotherapy by integrating complementary biological dimensions rather than relying on isolated surrogate markers (6, 16). However, greater analytical sophistication alone will not ensure clinical success. Predictive models must remain biologically grounded, reproducible, interpretable, and operationally feasible (228, 234). These considerations naturally raise the next translational question: which peripheral blood biomarkers are ready for clinical application today, and which remain investigational?

## 8 Clinical translation: what is ready, what is not?

Despite intense research activity surrounding peripheral blood biomarkers in PD-1/PD-L1 immunotherapy, clinical translation has progressed far more slowly than biomarker discovery (11, 12). A major reason is the tendency to extend exploratory associations into clinical decision-making before sufficient biological validation or prospective evidence has been established. Effective translation therefore requires a disciplined distinction between biomarkers that can meaningfully inform current clinical practice, those best suited for investigational use, and those that should not yet influence treatment selection (235). This distinction is essential to avoid conflating biological plausibility with clinical readiness.

### 8.1 Biomarkers with current or near-term clinical utility

Among currently available peripheral blood biomarkers, dynamic tumor-derived markers particularly ctDNA kinetics arguably offer the most immediate translational utility (12, 73). Early ctDNA decline or clearance following initiation of PD-1/PD-L1 blockade may provide clinically meaningful insight into treatment response, particularly in settings where radiographic interpretation is delayed, ambiguous, or confounded by pseudoprogression (236). Although ctDNA dynamics are fundamentally pharmacodynamic monitoring tools rather than pre-treatment predictive biomarkers, their integration into adaptive treatment assessment represents a realistic near-term application (237).

Routine laboratory indices such as ALC, NLR, PLR, CRP, and LDH may also offer pragmatic clinical value, primarily for contextual risk stratification rather than treatment selection (238). These markers can help frame baseline prognosis, identify patients with high systemic inflammatory burden or compromised physiological reserve, and inform supportive clinical interpretation (40, 239). However, their use should remain appropriately constrained; prognostic context should not be mistaken for evidence of treatment-specific resistance.

Composite biomarker approaches integrating accessible routine markers with more biologically specific assays may represent a feasible intermediate translational step, although broader validation remains necessary (224).

### 8.2 Biomarkers with strong rationale but insufficient clinical maturity

Several biomarker classes demonstrate strong biological rationale but remain insufficiently mature for routine clinical implementation. These include immune cell-derived biomarkers such as proliferative CD8^+^ T-cell signatures, functional exhaustion phenotypes, regulatory T-cell dynamics, MDSC profiling, and emerging monocyte-associated immune signatures (66, 240). Their mechanistic relevance to checkpoint biology is compelling, but interpretation remains highly context dependent and assay standardization is limited.

Similarly, emerging technologies including TCR repertoire analysis, EV biomarkers, transcriptomic immune signatures, and high-dimensional single-cell profiling currently offer substantial research value but remain primarily exploratory (241, 242). Their greatest near-term utility lies in biomarker-enriched clinical trials, translational immune monitoring studies, mechanistic stratification efforts, and adaptive trial design rather than immediate routine deployment (243).

In these contexts, peripheral blood biomarkers can serve as hypothesis-generating tools and may help refine future biomarker-guided therapeutic strategies.

### 8.3 Biomarkers that should not currently guide treatment decisions in isolation

A particularly important translational consideration is recognizing biomarkers that are frequently overinterpreted despite insufficient predictive specificity (244). Baseline inflammatory cytokines, acute-phase reactants, conventional serum tumor markers, and static global inflammatory ratios should not be used in isolation to determine eligibility for PD-1/PD-L1 therapy or to infer definitive treatment resistance (245).

Similarly, elevated baseline ctDNA or high tumor burden should not automatically be interpreted as evidence that checkpoint blockade will be ineffective (80). These biomarkers often reflect adverse prognostic biology rather than absence of immunotherapy responsiveness. Overreliance on such markers risks inappropriate exclusion of potentially responsive patients (34, 246).

This distinction is especially important as biomarker enthusiasm increasingly intersects with real-world clinical decision-making, where premature implementation of inadequately validated biomarkers could produce unintended harm (200).

### 8.4 Practical considerations for responsible implementation

For peripheral blood biomarkers to achieve meaningful clinical adoption, practicality must complement biological sophistication (11). Biomarkers intended for routine implementation should demonstrate reproducibility, interpretability, scalability, and compatibility with existing clinical workflows (18, 244). Highly complex assays that lack standardized analytical pipelines, validated analytical performance, or clear clinical decision thresholds are unlikely to gain widespread acceptance outside specialized research settings (247).

Equally important is clinician education and appropriate biomarker interpretation. Misclassification of prognostic biomarkers as predictive tools remains a recurring challenge in immunotherapy biomarker research and clinical practice (222). Clear communication regarding what a biomarker measures, the clinical question it is intended to address, and the limitation of its interpretation is essential for responsible integration into patient care (248). Without such clarity, even biologically informative biomarkers may contribute little to clinical decision-making.

Ultimately, successful translation will depend not simply on discovering increasingly sophisticated biomarkers, but on aligning biomarker biology with real-world clinical needs (244). Collectively, current evidence supports a cautious but constructive outlook for peripheral blood biomarkers in PD-1/PD-L1 immunotherapy (12). A limited number of applications, particularly dynamic treatment monitoring and contextual risk assessment, appear increasingly feasible, whereas most mechanistically attractive biomarkers remain investigational (249). Future progress will depend on more disciplined biomarker development strategies that distinguish predictive intent from prognostic association, prioritize prospective clinical validation, and balance biological sophistication with clinical interpretability, operational feasibility, and cost-effectiveness (18).

To facilitate practical interpretation, **Table 3** summarizes the current translational readiness of peripheral blood biomarker categories, their appropriate clinical applications, and major implementation considerations in PD-1/PD-L1 immunotherapy.

**Table 3:** Clinical readiness and translational considerations for peripheral blood biomarkers in PD-1/PD-L1 immunotherapy.

Biomarker category	Current clinical readiness	Appropriate use	Major caution	References
ctDNA kinetics	Near-term translational utility	Early response monitoring; adaptive assessment	Not a pre-treatment predictive biomarker	(250, 251)
Baseline ctDNA/tumor burden markers	Limited predictive utility	Prognostic risk assessment	High disease burden does not necessarily indicate immunotherapy resistance	(72, 246)
bTMB	Investigational/context dependent	Exploratory patient enrichment	Assay heterogeneity; tumor burden confounding	(252, 253)
CTCs	Exploratory	Molecular characterization; research use	Limited reproducibility	(254, 255)
ALC/NLR/PLR	Clinically accessible contextual biomarkers	Baseline risk stratification	Poor treatment specificity	(114, 256)
CRP/LDH/inflammatory markers	Contextual supportive biomarkers	Prognostic interpretation	Highly nonspecific inflammatory signals	(158, 257)
CD8^+^ T-cell dynamic immune signatures	Promising investigational biomarkers	Pharmacodynamic monitoring; prospective validation	Timing-dependent interpretation	(56, 258)
CD4^+^/Treg immune signatures	Exploratory	Immune mechanistic studies	Functional heterogeneity	(117, 259)
MDSC/suppressive myeloid signatures	Emerging investigational biomarkers	Resistance mechanism assessment	Poor phenotypic standardization	(132, 260)
TCR repertoire biomarkers	Emerging	Predictive biomarker development	Analytical complexity	(149, 261)
EV/exosomal biomarkers	Early-stage exploratory	Resistance biology; longitudinal monitoring	Technical standardization limitations	(176, 262)
Transcriptomic/single-cell immune profiling	Discovery-stage/translational research	Mechanistic stratification	High complexity; limited scalability	(263, 264)
Composite biomarker models	Promising but pre-validation	Integrated prediction frameworks	Reproducibility and implementation barriers	(18, 224)
ML predictive models	Experimental	Personalized decision support	Overfitting; interpretability concerns	(265, 266)

## 9 Future directions

The expanding literature on peripheral blood biomarkers in PD-1/PD-L1 immunotherapy reflects both scientific enthusiasm and persistent translational frustration. Despite numerous proposed biomarkers, few have achieved meaningful clinical implementation, largely because biomarker discovery has often outpaced mechanistic understanding, prospective validation, and practical clinical integration (12, 244). Future progress will require a shift away from descriptive association studies toward biologically grounded, hypothesis-driven biomarker development strategies explicitly aligned with clinical decision-making needs (267).

### 9.1 Redefining biomarker objectives: prediction, monitoring, or pharmacodynamic assessment?

A critical priority is greater conceptual clarity regarding biomarker intent. Many currently proposed biomarkers are discussed as predictive tools despite being more appropriately suited for prognostic stratification, treatment monitoring, or pharmacodynamic response assessment. This lack of definitional discipline has contributed substantially to interpretive confusion and translational stagnation (213).

Future biomarker development should explicitly distinguish whether a biomarker is intended to guide pre-treatment therapeutic selection, monitor early treatment response, identify emerging resistance, or assess immune activation during therapy. Aligning biomarker design with its intended clinical function will improve study design, endpoint selection, and translational relevance (268).

### 9.2 Moving from single biomarkers to biologically integrated frameworks

The biological complexity of checkpoint inhibitor responsiveness strongly argues against continued reliance on isolated biomarker strategies. Future predictive models will likely require integration of complementary biological dimensions, including tumor burden, systemic immune competence, inflammatory context, immune repertoire dynamics, and treatment-induced biological adaptation (269, 270).

Importantly, integration should be biologically rational rather than purely data-driven. Combining biomarkers without mechanistic coherence risks generating statistically attractive but clinically fragile models. The most successful future frameworks will likely balance multidimensional complexity with biological interpretability and clinical practicality (271).

### 9.3 Expanding systems-level immune monitoring

Emerging technologies capable of high-resolution systemic immune profiling will likely play an increasingly important role in future biomarker development. TCR repertoire analysis, circulating transcriptomics, EV profiling, single-cell immune phenotyping, and other multi-omic approaches offer opportunities to move beyond conventional surrogate biomarkers toward mechanistically richer characterization of treatment responsiveness (11).

These approaches may be particularly valuable for identifying dynamic immune trajectories associated with response, resistance, or immune-related toxicity. However, technological sophistication alone will not guarantee translational success. Standardization, reproducibility, interpretability, and cost-effectiveness must remain central considerations (190).

### 9.4 Prospective validation and biomarker-embedded clinical trial design

A major limitation of the current literature is the predominance of retrospective analyses and exploratory biomarker associations. Future progress will require prospective biomarker validation within rigorously designed clinical trials that predefined biomarker hypotheses, analytical methodologies, sampling schedules, and clinically meaningful endpoints (272).

Biomarker-embedded adaptive trial designs may be particularly valuable, enabling real-time refinement of therapeutic strategies based on emerging biological response patterns. Such approaches could accelerate transition from exploratory biomarker observation to actionable clinical implementation (243).

### 9.5 Artificial intelligence and computational integration with caution

Artificial intelligence (AI) and machine learning (ML) approaches will likely become increasingly important as multidimensional biomarker datasets expand. Computational integration may improve predictive precision by identifying complex nonlinear biological relationships that are not easily captured through conventional analyses (273).

However, future computational models must prioritize transparency, interpretability, and external validation. Highly complex predictive algorithms that function as biological black boxes may offer limited clinical trust or operational feasibility. The goal should not be algorithmic complexity for its own sake, but clinically meaningful decision support grounded in biological plausibility.

Clinical translation requires validation across independent and diverse datasets representing different tumor types, disease stages, demographic and ancestral backgrounds, comorbidities, treatment regimens, geographic regions, and laboratory platforms. Missing-data handling, biomarker sampling windows, outcome definitions, and analytical procedures should be prespecified. Model evaluation should extend beyond discrimination to include calibration, clinical net benefit, subgroup performance, and robustness to temporal or institutional dataset shifts. Particular attention should be given to fairness across clinically relevant patient subgroups and to determining whether predictions are driven by treatment-specific biology or by general prognostic factors such as tumor burden, performance status, or systemic inflammation (216, 274).

Operational implementation further requires a locked model and predefined decision thresholds, reproducible biomarker assays, integration with electronic health records and routine clinical workflows, acceptable turnaround time and cost, and clinician-facing explanations that support rather than obscure clinical judgment. Appropriate regulatory oversight and post-deployment monitoring are also necessary to detect performance drift, changes in data quality, and inequitable outcomes. Ultimately, clinical utility should be established through prospective impact studies demonstrating that AI/ML-guided decisions improve patient outcomes, reduce ineffective treatment, or provide other meaningful benefits compared with standard clinical assessment (275, 276).

### 9.6 Toward biomarker-guided precision immunotherapy

Ultimately, the future of peripheral blood biomarkers lies not in identifying a single universal predictive marker, but in developing biologically coherent frameworks capable of supporting precision immunotherapy across diverse clinical contexts. Such frameworks should distinguish predictive biology from prognostic background noise, incorporate dynamic treatment evolution, and remain adaptable to disease-specific biological heterogeneity (277).

Success will require close integration between immunobiology, translational oncology, biomarker science, computational modeling, and pragmatic clinical implementation. Only through this multidisciplinary alignment can peripheral blood biomarkers evolve from exploratory correlates into reliable tools for biomarker-guided immunotherapy (11).

Collectively, future progress will depend less on expanding biomarker quantity and more on improving biomarker quality, mechanistic clarity, and translational discipline (244). These priorities are essential for transforming promising biological observations into clinically actionable strategies. At the same time, interpretation of the current evidence must remain grounded in recognition of important methodological and conceptual limitations (202, 278).

**Figure 3:**
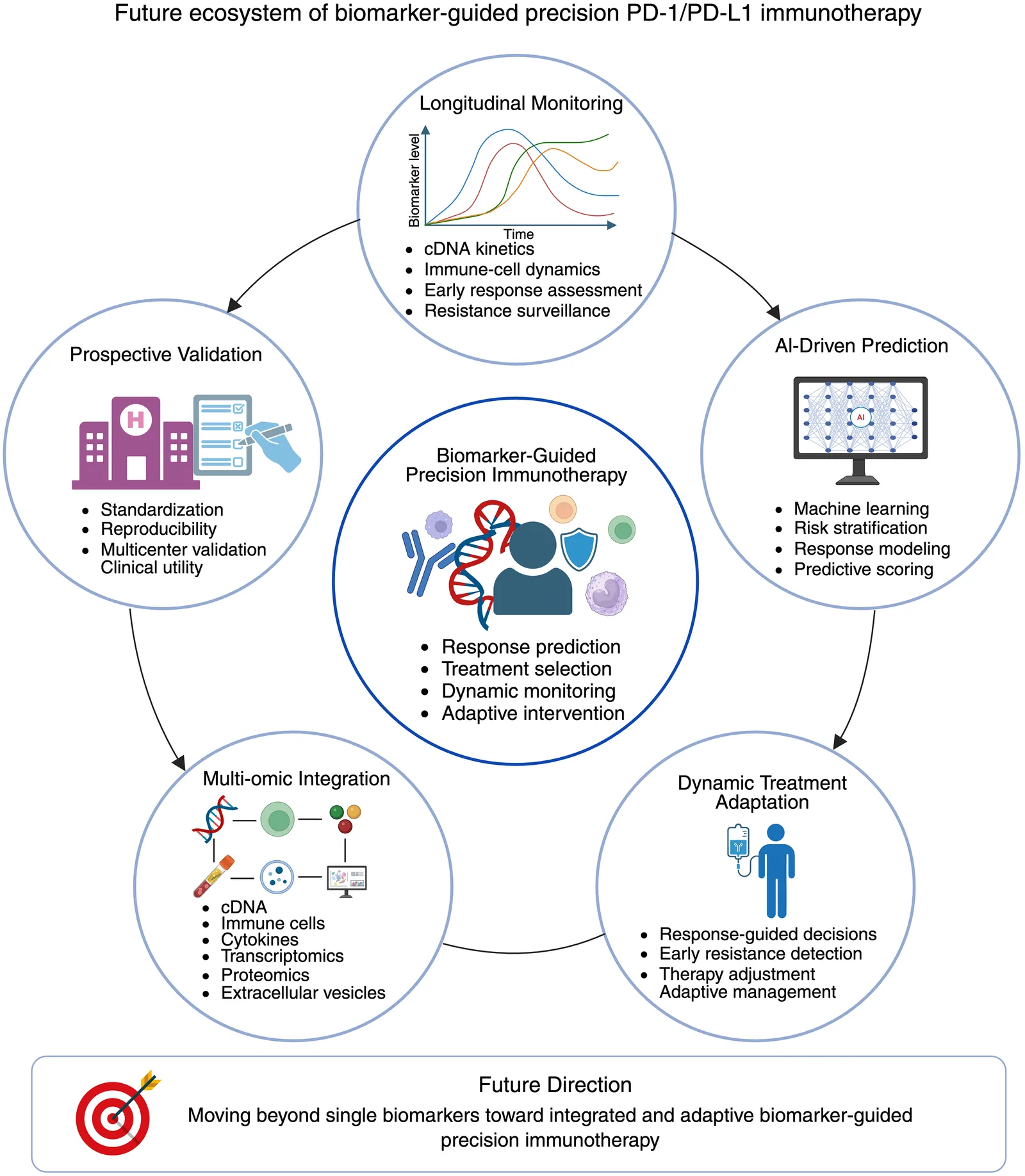
Future ecosystem of biomarker-guided precision PD-1/PD-L1 immunotherapy. Emerging biomarker strategies are increasingly moving beyond single-marker approaches toward integrated, dynamic, and clinically actionable frameworks for precision immunotherapy. At the center of this ecosystem is biomarker-guided precision immunotherapy, which aims to support response prediction, treatment selection, dynamic monitoring, and adaptive intervention. Five interconnected domains contribute to this framework. Longitudinal monitoring captures temporal changes in circulating biomarkers, including ctDNA kinetics and immune-cell dynamics, enabling early response assessment and resistance surveillance. AI-driven prediction integrates complex biomarker datasets to improve risk stratification, response modeling, and predictive scoring. Dynamic treatment adaptation facilitates biomarker-informed therapeutic adjustment based on evolving response patterns and emerging resistance mechanisms. Multi-omic integration combines complementary biological information from ctDNA, immune cells, cytokines, transcriptomic profiles, proteomic signatures, and EVs to generate a more comprehensive representation of tumor-immune interactions. Prospective validation establishes clinical utility through standardization, reproducibility, multicenter validation, and rigorous assessment of clinical benefit. Together, these interconnected components define a future paradigm in which integrated biomarker ecosystems support adaptive and personalized PD-1/PD-L1 immunotherapy.

To summarize the strategic path toward clinically actionable biomarker-guided immunotherapy, **Figure 3** outlines a translational roadmap from biomarker discovery to precision implementation in PD-1/PD-L1 blockade.

## 10 Limitations of current evidence

Interpretation of the current literature on peripheral blood biomarkers in PD-1/PD-L1 immunotherapy requires important caution. Although numerous biomarkers have been associated with treatment outcomes, the overall evidence base remains constrained by substantial conceptual, methodological, and translational limitations that complicate clinical interpretation and hinder implementation (18, 222).

A major limitation is the persistent conflation of prognostic and predictive biomarker functions (21, 233). Many studies report associations between circulating biomarkers and survival outcomes without adequately establishing treatment-specific predictive relevance. Because systemic biomarkers are strongly influenced by tumor burden, host physiological reserve, chronic inflammation, prior therapies, and comorbid biological processes, observed associations may reflect general disease biology rather than genuine immunotherapy responsiveness. This interpretive ambiguity remains one of the most significant barriers to biomarker translation.

Methodological heterogeneity further limits comparability across studies (12, 279). Variability in assay platforms, sample processing protocols, biomarker definitions, cut-off thresholds, timing of blood collection, treatment regimens, and endpoint selection has generated substantial inter-study inconsistency. These challenges are particularly pronounced for emerging biomarker platforms such as immune repertoire analysis, EV profiling, transcriptomic signatures, and high-dimensional immune phenotyping, where technical standardization remains limited.

The predominance of retrospective and exploratory study designs represents another important limitation (280). Many reported biomarkers have been identified through hypothesis-generating analyses without prospective validation, increasing vulnerability to selection bias, overfitting, false discovery, and limited reproducibility (267). Small sample sizes and heterogeneous patient populations further constrain generalizability, particularly when biomarker performance may vary substantially across tumor types, disease stages, and treatment combinations (6, 281).

An additional challenge lies in the biological disconnect between peripheral circulation and the TME (282). Although peripheral blood offers practical accessibility and dynamic monitoring potential, circulating biomarkers may not fully recapitulate local tumor-immune interactions that directly govern checkpoint inhibitor responsiveness. Peripheral immune signatures may therefore provide incomplete or context-dependent representations of biologically relevant antitumor immunity.

Finally, rapid technological innovation has introduced increasing analytical complexity without necessarily improving clinical interpretability (283). More sophisticated biomarker platforms are not inherently more clinically actionable. Without clear biological rationale, reproducibility, and pragmatic implementation pathways, highly complex biomarker signatures risk remaining academically informative but clinically impractical.

Collectively, these limitations underscore the need for more disciplined biomarker development frameworks that integrate mechanistic understanding, methodological rigor, prospective validation, and clinical practicality (18, 284).

## 11 Conclusions

**Figure 4:**
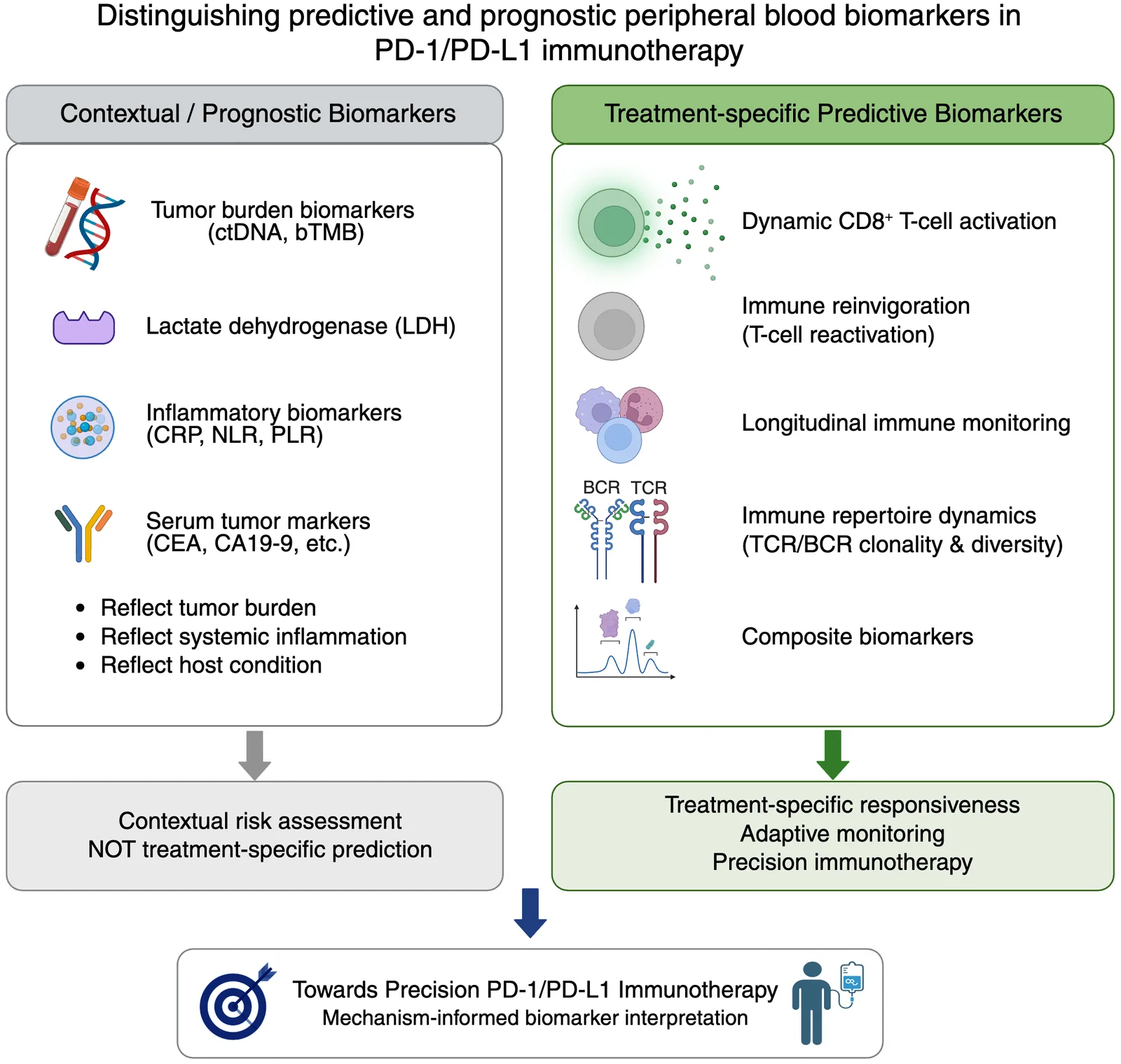
Distinguishing contextual/prognostic from treatment-specific predictive peripheral blood biomarkers in PD-1/PD-L1 immunotherapy. Contextual biomarkers primarily support risk assessment, whereas treatmentspecific predictive biomarkers may enable precision immunotherapy.

Peripheral blood biomarkers offer an attractive and increasingly important avenue for advancing precision immunotherapy in patients receiving PD-1/PD-L1 blockade, owing to their minimally invasive accessibility, feasibility for serial monitoring, and potential to capture dynamic systemic biological responses during treatment (12). However, enthusiasm for their clinical utility has often outpaced careful biological interpretation. A recurring challenge across the current literature is the failure to distinguish biomarkers that primarily reflect prognostic disease context from those that genuinely provide treatment-specific predictive insight (**Figure 4**).

This review highlights that many widely studied circulating biomarkers, including tumor burden-associated markers, inflammatory indices, and soluble mediators, provide valuable contextual and monitoring information but offer limited standalone predictive specificity for checkpoint inhibitor responsiveness. In contrast, immune cell-derived biomarkers and emerging high-dimensional immune-monitoring approaches demonstrate stronger mechanistic relevance to treatment efficacy, although their clinical applicability remains constrained by biological complexity, methodological heterogeneity, and limited prospective validation.

At present, the most immediate clinical value of peripheral blood biomarkers lies in dynamic treatment monitoring and contextual risk assessment, whereas robust treatment-selection biomarkers for PD-1/PD-L1 blockade remain an unmet need.

First, the intended clinical use prognostic assessment, pretreatment selection, early response monitoring, resistance detection, or toxicity prediction should be defined before biomarker development. Second, biomarker assays, sample processing procedures, collection schedules, thresholds, and statistical analysis plans should be standardized and prospectively specified. Third, claims of treatment-specific predictive utility should be supported by randomized treatment-by-biomarker interaction testing, whereas composite and AI/ML models require rigorous independent external validation. Fourth, development should prioritize parsimonious, biologically coherent combinations that integrate complementary baseline and longitudinal information without introducing unnecessary redundancy or complexity. Finally, prospective impact studies must demonstrate that biomarker-guided decisions provide clinical utility, operational feasibility, cost-effectiveness, and equitable performance across patient populations. Until these requirements are met, peripheral blood biomarkers should complement rather than replace integrated clinical and tissue-based assessment in PD-1/PD-L1 immunotherapy.
